# Dupilumab in real-world pediatric asthma: Enhanced control and quality of life for children and caregivers^☆^

**DOI:** 10.1016/j.waojou.2026.101251

**Published:** 2026-01-18

**Authors:** Ricardo Martínez-Tenopala, Jimena Prieto-Gomez, María Julia Rendón-Salazar, Tamara Hernández-Hernández, Javier Zermeño-Vallet, Diego Mauricio Gómez-González, Carlos Andrés Gómez-Nuñez, Luis Ángel Hernández-Zárate, Víctor Gonzalez-Uribe

**Affiliations:** a AlergiaMx: Clinical and Research Center, Mexico City, Mexico; b Facultad Mexicana de Medicina, Universidad La Salle México, Mexico City, Mexico; c Hospital Ángeles del Pedregal, Mexico City, Mexico

**Keywords:** Dupilumab, Asthma, Child, Quality of life, Type 2 inflammation, Biological therapy

## Abstract

**Background:**

Dupilumab has demonstrated therapeutic benefits and improved quality of life (QoL) in children with uncontrolled asthma. This real-world study—the first in a Latin-American pediatric population—evaluated its impact on asthma control and QoL in children with moderate-to-severe uncontrolled type 2 asthma and their caregivers.

**Methods:**

We conducted a retrospective observational study of patients aged 4–16 years with moderate-to-severe uncontrolled type 2 asthma (blood eosinophils ≥150 cells/μL or fractional exhaled nitric oxide ≥20 ppb). Eleven patients received dupilumab and 12 received conventional inhaled therapy, with 52-week follow-up. Asthma control was assessed using the 7-item Asthma Control Questionnaire interviewer-administered version (ACQ-7-IA). QoL was evaluated using the Pediatric Asthma Quality of Life Questionnaire interviewer-administered version (PAQLQ[S]-IA) and the Pediatric Asthma Caregiver's Quality of Life Questionnaire (PACQLQ). The primary endpoints were changes in ACQ-7-IA, PAQLQ(S)-IA, and PACQLQ scores, and the proportion achieving clinically significant improvement (≥0.5 points).

**Results:**

Dupilumab significantly improved ACQ-7-IA scores from week 4, sustained through week 52 (least squares mean difference −0.44, 95% CI −0.59 to −0.30; p < 0.001). At week 52, 86% of dupilumab-treated patients achieved clinically significant improvement versus 75% with conventional therapy (p = 0.041). Well-controlled asthma (ACQ-7-IA ≤0.75) was achieved in 73% versus 36% (p < 0.003). Dupilumab also improved PAQLQ(S)-IA and PACQLQ scores from week 24, with 100% of patients and caregivers achieving clinically significant improvement at week 52.

**Conclusions:**

In Latin-American children with moderate-to-severe type 2 asthma, dupilumab was associated with early and sustained improvements in asthma control and QoL for both patients and caregivers, supporting its role as an effective therapeutic option in real-world pediatric practice.

## Introduction

Asthma is among the most prevalent chronic diseases worldwide, affecting hundreds of millions of individuals, many of whom remain undiagnosed or inadequately treated.^1^ In a Latin American country, approximately 8.5 million people are reported to live with asthma—a figure consistent with recent global estimates indicating a prevalence of around 9.1% in children, 11% in adolescents, and 6.6% in adults.^2^^,^^3^

It is estimated that between one-third and one-half of these patients have poor disease control. This is associated with a substantial decline in health-related quality of life (HRQoL), a marked increase in healthcare expenditures, a greater burden on parents and caregivers, and ongoing risk, as uncontrolled asthma remains a major cause of hospitalization.^1^

Although standard treatment guidelines are well established, a substantial proportion of patients continue to present severe symptoms or inadequate asthma control. This persistent disease burden underscores the need to investigate alternative therapeutic strategies, particularly biological agents. Biologic therapies have shown substantial efficacy in the management of severe asthma and are widely used as adjunctive treatments for pediatric patients with uncontrolled or moderate-to-severe disease.^4^

Type 2 inflammation represents the most common asthma phenotype in the pediatric population.^5^ Dupilumab is a humanized monoclonal antibody specifically developed to treat diseases characterized by type 2 inflammation, particularly asthma. Its mechanism of action involves blocking the α-subunit of the shared receptor for interleukins IL-4 and IL-13, thereby inhibiting both Th2 signaling pathways implicated in airway inflammation, bronchial hyperresponsiveness, and excessive mucus production.^6^ Dupilumab has been approved by local regulatory authorities for the treatment of children aged 6 years and older with specific forms of moderate-to-severe asthma.^7^

Although effective tools have been developed to assess health-related quality of life (HRQoL), a common need remains to understand this dimension across more diverse populations.^8^ This need is particularly relevant in pediatric populations, where asthma affects not only the quality of life of children but also their families and caregivers, who face a substantial emotional and functional burden.^9^, ^10^, ^11^, ^12^ In light of these considerations, the present study aims to examine these aspects in children with asthma receiving biological therapy with dupilumab.

## Materials and methods

We conducted a real-world, retrospective observational study of children aged 4–16 years with uncontrolled moderate-to-severe asthma according to the Global Initiative for Asthma (GINA) guidelines,^1^ at a referral center in a Latin American country, with a 1-year treatment follow-up. Data was collected over 4 years (March 2020 to January 2024). Patients received either dupilumab or inhaled therapy as part of routine follow-up following GINA recommendations.

Two populations were analyzed: patients with type 2 asthma (defined by baseline blood eosinophils ≥150 cells/μL or fractional exhaled nitric oxide ≥20 ppb), and a subpopulation of type 2 asthma patients without access to biologic therapy, who were managed with inhaled corticosteroids and long-acting β2-agonists (ICS/LABA) or triple therapy with ICS/LABA plus long-acting muscarinic antagonist (LAMA), corresponding to GINA step III or IV management.

Patients were treated according to authorized prescribing recommendations by local regulatory authorities, with follow-up visits every 2 weeks for 52 weeks. All patients continued their baseline ICS/LABA treatment.

Clinicians administered all questionnaires to the children, with assistance from parents or caregivers. Asthma control was evaluated using the 7-item interviewer-administered Asthma Control Questionnaire (ACQ-7-IA), designed to assess its impact on health-related quality of life (HRQoL). This questionnaire includes 5 items on asthma symptoms, 1 item on medication use (daily bronchodilator use), and 1 item on percent predicted forced expiratory volume in 1 s (FEV1).

Asthma-specific HRQoL, encompassing symptoms, activity limitation, and emotional functioning domains in children was assessed using the interviewer-administered Pediatric Asthma Quality of Life Questionnaire (PAQLQ(S)-IA). All instruments have been validated in children with asthma or their caregivers.^2^^,^^3^ PAQLQ(S)-IA and the Pediatric Asthma Caregiver's Quality of Life Questionnaire (PACQLQ) were evaluated in children aged ≥7 years and their caregivers, respectively.

For ACQ-7-IA, evaluation criteria included change from baseline in overall ACQ-7-IA score and item scores over time. A clinically significant response was defined as an improvement from baseline of ≥0.5 points in the ACQ-7-IA score.^4^ At weeks 24 and 52, the proportion of patients achieving well-controlled asthma (ACQ-7-IA and ACQ-5-IA ≤0.75 points), adequately controlled disease (ACQ-7-IA and ACQ-5-IA <1 point), and controlled disease (ACQ-7-IA and ACQ-5-IA <1.5 points) was also assessed.^5^

For PAQLQ(S)-IA and PACQLQ, changes from baseline in global scores and in emotional function and activity limitation domains were assessed at weeks 24 and 52; changes in the symptom domain score for PAQLQ(S)-IA were also evaluated at these time points.^6^^,^^7^ Changes from baseline in PRQLQ-IA scores at weeks 24 and 52 were assessed in children with a medical history of allergic rhinitis. Clinically significant responses in PAQLQ(S)-IA and PRQLQ-IA were defined as an improvement from baseline of ≥0.5 points in the global score.^8^

## Ethics and consent

Written informed consent was obtained from all participants or their legal guardians. This was a retrospective observational study and therefore was not subject to clinical trial registration requirements.

## Statistical analysis

Changes from baseline in ACQ-7-IA item scores, as well as in overall PACQLQ and PAQLQ(S)-IA scores over time, were analyzed. To compare the proportion of children achieving a clinically meaningful improvement, a difference-in-proportions test was used to compare the treatment groups. To further assess the independent effect of dupilumab on asthma control, we fitted multivariable linear regression models with change in ACQ-7 score (final – baseline) as the dependent variable. Covariates included baseline ACQ-7, blood eosinophils, FeNO, and IgE levels, with treatment group (dupilumab vs conventional therapy) as the main exposure variable. Given the evaluation of multiple outcomes (ACQ-7, PAQLQ, PACQLQ), p-values for secondary endpoints were adjusted using the Benjamini–Hochberg procedure to control the false discovery rate (FDR). A q-value <0.05 was considered statistically significant. All tests were conducted using a two-sided significance level of 0.05.

## Results

### Patients

Of the 26 patients included, 23 (88%) had type 2 asthma at study baseline, and 16 (61%) had blood eosinophil counts ≥300 cells/μL. Most children (>87%) were white, and approximately two-thirds were male. In both populations, baseline global scores for the ACQ-5/7-IA and PAQLQ(S)-IA were comparable between the treatment groups (dupilumab vs conventional inhaled therapy). Across all groups, 78–88% of patients had a history of comorbid allergic rhinitis (Table 1).

**Table 1:** Baseline characteristics by treatment group

Variable	Dupilumab Group (n = 11)	Conventional Group (n = 10)	p-value
**Demographics**			
Age (years), mean (95% CI)	11.5 (9.6–13.4)	10.8 (8.7–12.9)	0.29
Sex (male), n (%)	6 (54.5%)	5 (50.0%)	1.00
**Clinical history & comorbidities**			
Exacerbations (prev. year), mean ± SD	5.09 ± 1.30	5.10 ± 0.99	0.98
Allergic rhinitis, n (%)	11 (100%)	10 (100%)	1.00
Atopic dermatitis, n (%)	3 (27.3%)	2 (20.0%)	1.00
Obesity/Overweight, n (%)	0 (0%)	1 (10.0%)	0.47
Food allergy, n (%)	0 (0%)	1 (10.0%)	0.47
**Biomarkers of T2 inflammation**			
Blood eosinophils (cells/μL)	717.7 ± 175.3	731.0 ± 172.8	0.86
FeNO (ppb)	71.0 ± 16.7	48.2 ± 16.8	<0.01
Total IgE (IU/mL)	598.7 ± 325.0	611.6 ± 322.0	0.93
**Lung function (spirometry)**			
Pre-BD FEV_1_ (%)	75.39 ± 5.25	72.34 ± 6.64	0.24
FEV_1_/FVC ratio	0.73 ± 0.05	0.71 ± 0.03	0.42
**Patient-reported outcomes**			
ACQ-7 score	2.68 ± 0.39	2.58 ± 0.41	0.59
PAQLQ score	3.80 ± 0.46	3.82 ± 0.56	0.96
PACQLQ score (caregivers)	3.73 ± 0.41	3.95 ± 0.45	0.27

### Asthma control

In the type 2 asthma population, treatment with dupilumab was associated with a significantly greater improvement in asthma control, as measured by the ACQ-7-IA questionnaire, compared to conventional inhaled therapy.

**Fig. 1:**
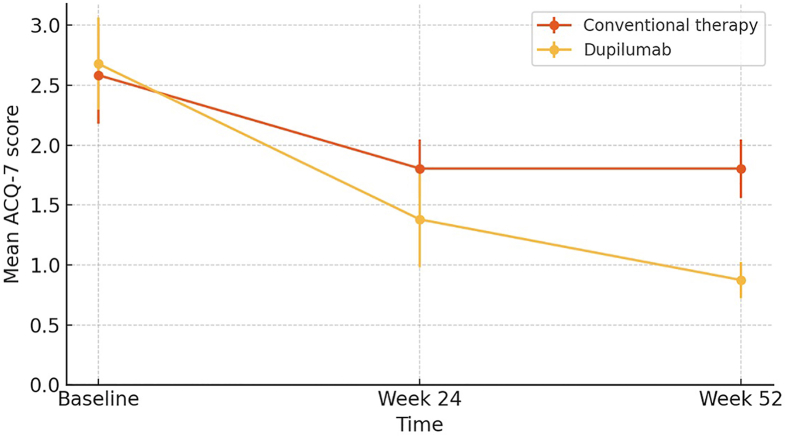
Evolution of asthma control (ACQ-7) over time. *Asthma control progression measured with the ACQ-7, comparing dupilumab vsconventional inhaled therapy*

Cross-sectional analysis between groups (dupilumab vs inhaled therapy) showed a greater mean difference favoring dupilumab of −0.43 points at week 24 (95% CI: −0.71 to −0.14; p = 0.0089) and −0.93 points at week 52 (95% CI: −1.11 to −0.76; p < 0.0001). Additionally, a higher proportion of patients in the dupilumab group achieved a clinically significant improvement (≥0.5 points) at the end of the year (100% vs 90%, p = 0.476) (Fig. 1). Significant improvements in ACQ-7-IA item scores were observed from baseline for dupilumab compared to inhaled therapy in all items except item 1 (asthma-related nocturnal awakenings) at week 24, and in all items at week 52.

Clinical asthma control was evaluated using established ACQ-7-IA cutoff points. At week 24, no patient in either group reached the threshold for well-controlled asthma (ACQ-7 ≤ 0.75). Eighteen-point two percent of children treated with dupilumab achieved adequate control (ACQ-7 < 1.00), whereas no patients in the inhaled therapy group reached this control level. At week 52, 18% of dupilumab-treated patients attained well-controlled asthma, and 72.7% achieved adequate control. None of the patients in the conventional therapy group reached acceptable or optimal clinical control.

Longitudinal analysis showed that patients receiving dupilumab had a mean reduction of −1.81 points in ACQ-7 score from baseline to week 52 (95% CI: −2.01 to −1.60; p < 0.0001), while the conventional inhaled therapy group showed a mean reduction of −0.78 points (95% CI: −0.93 to −0.63; p < 0.0001) (Fig. 1).

Regarding asthma exacerbations, by week 52, a significantly greater proportion of patients treated with dupilumab experienced no exacerbations compared to the conventional inhaled therapy group (81.8% vs 0.0%); 100% of patients in the inhaled therapy group had at least 1 exacerbation, compared to 18.2% in the dupilumab group.

In the multivariate linear regression model, including treatment group, baseline FeNO, eosinophils, and IgE as covariates, dupilumab treatment was the only statistically significant predictor, associated with a mean reduction of −0.96 points in ACQ-7 score compared to conventional therapy (95% CI: −1.20 to −0.71; p < 0.001). Biomarkers showed no significant associations with final ACQ-7 scores.

In an expanded multivariable model additionally adjusting for baseline ACQ-7, dupilumab remained significantly associated with greater improvement in asthma control (β = −0.92; 95% CI −1.11 to −0.73; p < 0.001).

### Patient quality of life (PAQLQ)

Regarding patient quality of life assessed by the PAQLQ, the dupilumab group was associated with significantly greater improvements compared to the conventional therapy group.

Cross-sectional analysis between groups (dupilumab vs inhaled therapy) showed a greater mean difference favoring dupilumab at week 24 (+1.46 points) and week 52 (+1.64 points), both statistically significant (p < 0.0001). Similar to the ACQ-7 evaluation, a higher proportion of patients in the dupilumab group achieved a clinically significant improvement (≥0.5 points) by the end of the year (100% vs 70%; p = 0.0902).

**Fig. 2:**
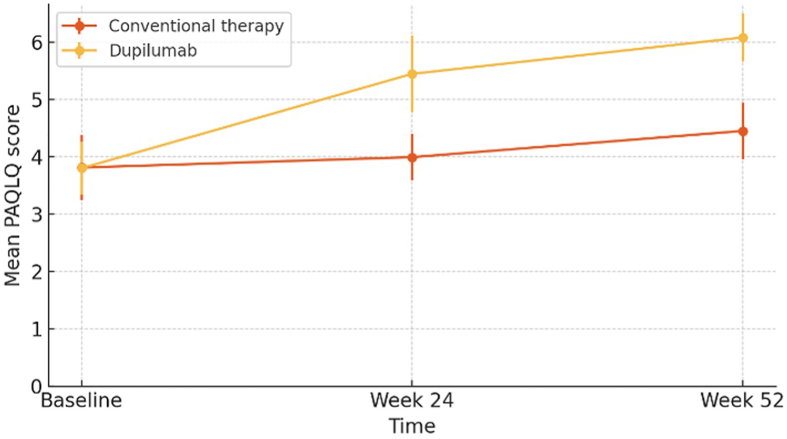
Evolution of patient quality of life (PAQLQ) over time. *Progression of patient QoL assessed using the PAQLQ questionnaire in both groups*

In the longitudinal analysis, the dupilumab group showed an absolute difference of +1.65 points at week 24 and + 2.28 points at week 52 (p < 0.0001), while the inhaled therapy group showed increases of +0.18 points at week 24 (p = 0.107) and +0.64 points at week 52 (p < 0.0001) (Fig. 2). After correction for multiple comparisons using the Benjamini–Hochberg procedure, improvements in PAQLQ remained statistically significant (q < 0.05).

### Caregiver quality of life (PACQLQ)

Regarding caregiver quality of life, assessed using the PACQLQ questionnaire, the dupilumab-treated group showed greater improvements compared to the conventional inhaled therapy group.

At week 24, a mean difference of +0.12 points favoring dupilumab (3.81 vs 3.69) was observed, which did not reach statistical significance (p = 0.5733). At week 52, the difference between groups was statistically significant, with a mean PACQLQ score of 5.20 versus 4.52, representing a mean difference of +0.70 points (95% CI: 0.26 to 1.15; p = 0.0065) in favor of dupilumab. At the end of the study (week 52), 100% of caregivers in the dupilumab group achieved a clinically significant improvement compared to 70% in the conventional therapy group (p = 0.0902).

**Fig. 3:**
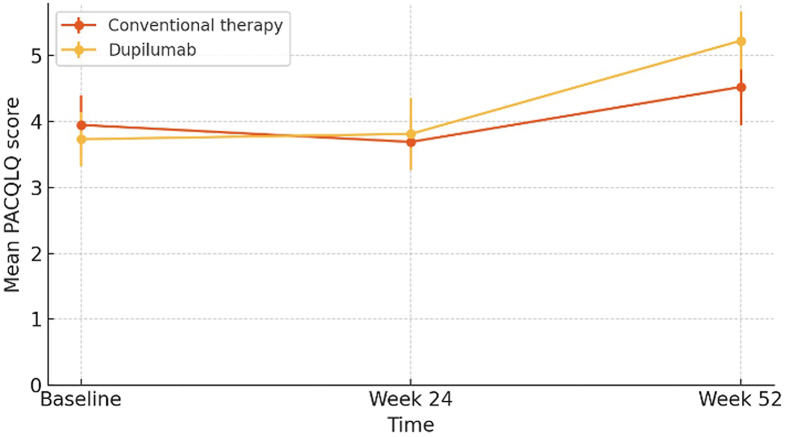
Evolution of caregiver quality of life (PACQLQ) over time. *Progression of caregiver QoL assessed using the PACQLQ questionnaire in both groups*

**Fig. 4:**
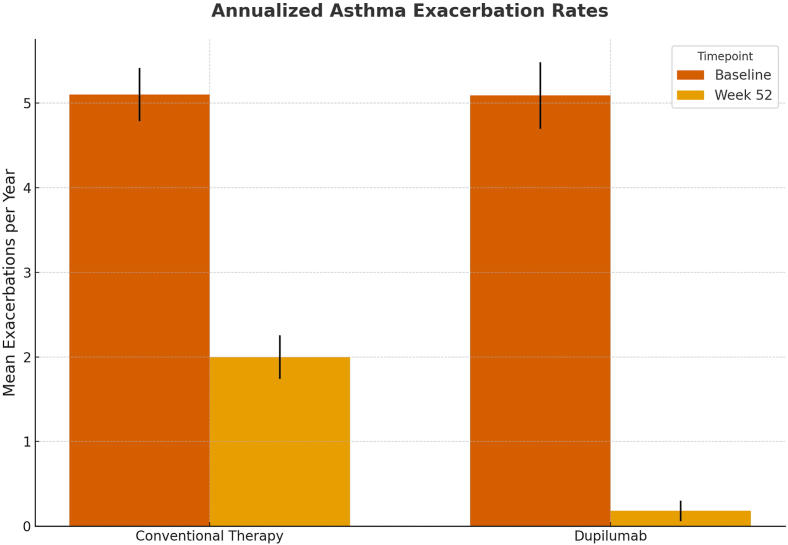
**Annualized asthma exacerbation rates at baseline and week 52.** Mean annualized asthma exacerbation rates comparing dupilumab and conventional inhaled therapy at baseline and after 52 weeks of follow-up. Error bars represent standard deviation

In the dupilumab-treated group, change from baseline to week 52 showed a significant improvement in PACQLQ score (+1.50 ± 0.32 points; p < 0.0001). In contrast, the conventional inhaled therapy group also demonstrated a significant improvement at week 52, albeit of lesser magnitude (+0.58 ± 0.16 points). The between-group difference in change was statistically significant (p < 0.0001) (Fig. 3). Similarly, caregiver quality of life improvements remained statistically significant after FDR adjustment (q < 0.05), confirming robustness of the findings across multiple outcomes (see Fig. 4).

## Discussion

### Disease control

In this real-world study of a pediatric cohort with uncontrolled moderate-to-severe type 2 asthma—defined by serum eosinophil counts ≥150 cells/μL or fractional exhaled nitric oxide ≥20 ppb—treatment with dupilumab provided significantly superior symptomatic control compared to conventional inhaled therapy. These benefits were observed in early stages and were sustained and progressive throughout the 52-week follow-up period.

Our findings align with international reports demonstrating a significant reduction in the annualized exacerbation rate in patients treated with dupilumab versus conventional therapy 13, 14, 15, 16, 17.

It is noteworthy that in our cohort, no patient treated with conventional therapy remained free of exacerbations, highlighting the efficacy of dupilumab treatment. These data reinforce the evidence supporting dupilumab as a disease-modifying agent by substantially reducing the exacerbation burden in pediatric patients with moderate-to-severe type 2 asthma. In the pediatric VOYAGE trial by Bacharier et al., which included 408 children aged 6–11 years, a significant reduction in the annualized exacerbation rate was reported, with 77.9% of patients remaining exacerbation-free during dupilumab treatment—comparable to the 81.8% observed in our cohort, slightly exceeding previous results.^18^

Regarding symptomatic control, our results are consistent with previous studies demonstrating significant improvements in ACQ and other assessment scales following dupilumab use. The dupilumab group showed a mean reduction of −1.81 points in ACQ-7, with a difference of 0.93 points compared to conventional therapy, reflecting a pronounced improvement in asthma control and exceeding the clinically relevant threshold of 0.5 points in the ACQ. This threshold is not only statistically significant, but also considered sufficient to change patient classification and therapeutic decisions.^19^ In the QUEST trial, involving patients older than 12 years treated with dupilumab, improvements in lung function and decreases in ACQ-5 scores were observed from week 2 and present throughout 52 weeks,^13^ where the mean reduction in ACQ-5 among dupilumab-treated patients was −1.39 points after 52 weeks, with a difference of 0.28 points compared to conventional therapy.^17^

In our study, the mean reduction in ACQ-7 was −0.93 points over the same period, indicating a favorable effect in our cohort. Although our analysis did not include biomarkers as in other studies, the improvements observed in ACQ-7-IA scores reflect positive symptomatic control and indicate the benefit obtained in lung function. Similar magnitude results have been reported in the literature; the VENTURE trial reported improvements in ACQ-5 at week 24, with a mean difference of −0.47 points favoring dupilumab compared to conventional therapy (vs −0.43 points in our study).^15^ These findings were confirmed in the TRAVERSE extension study, reported by Sher et al., where benefits were sustained up to 148 weeks.^20^^,^^21^ In the pediatric population, VOYAGE demonstrated that dupilumab treatment significantly improved FEV1 values and asthma control via ACQ-7, with a difference of −0.44 points compared to conventional therapy at week 52, a value close to ours, reinforcing the consistency of findings.^22^

To complement this evidence, real-world studies in other populations, such as that by Shi T. et al. in China with 28 pediatric patients, reported results consistent with ours: reduction in exacerbations and improved symptomatic asthma control, evidenced by increases in ACT/C-ACT scores.^23^ This reinforces the robustness of dupilumab's benefits and suggests that its effects are maintained across diverse populations and routine clinical settings.

### Patient quality of life

Health-related quality of life (HRQoL) is a fundamental component in the comprehensive evaluation of pediatric asthma.^24^

In our study, patients treated with dupilumab showed a significant improvement in HRQoL measured by PAQLQ, with an average increase of 1.46 points at 24 weeks and 1.64 points at 52 weeks. Furthermore, 100% of patients in this group achieved a clinically relevant improvement (≥0.5 points) by the end of the year, compared to 70% in the conventional therapy group. These results are consistent with those reported in the VOYAGE trial, where dupilumab significantly improved HRQoL, reflected in PAQLQ(S)-IA scores at weeks 24 and 52.^18^^,^^25^

In adults, studies have also demonstrated that dupilumab produces significant clinical improvements in asthma control, symptoms, quality of life, and productivity, as reflected in AQLQ scores.^26^

### Caregiver quality of life

Caregiver quality of life is crucial as it influences treatment adherence and family well-being.^27^ In our study, assessed by the PACQLQ questionnaire, caregiver quality of life was superior in the dupilumab-treated group compared to the conventional therapy group. At 24 weeks, the mean difference between groups was 0.13 points, not reaching statistical significance; however, at 52 weeks, the mean difference was statistically significant (0.70 points) in favor of dupilumab.^18^^,^^28^ Furthermore, by the end of the year, 100% of caregivers in the dupilumab group achieved a clinically significant improvement, compared to 70% in the conventional therapy group. These results suggest that dupilumab's impact on caregiver quality of life has important implications for treatment adherence and the family environment. Previous studies support this evidence: in VOYAGE, the mean difference in PACQLQ was 0.25 points at week 24 and 0.47 points at week 52, favoring dupilumab.^18^^,^^28^ In both studies, improvements at week 52 were significantly greater among caregivers of patients treated with dupilumab.^27^

Together, these findings reinforce the clinical value of dupilumab treatment, supporting it as an effective therapeutic alternative in the comprehensive management of moderate-to-severe type 2 asthma in the pediatric population. Our study contributes to this body of evidence by demonstrating that, in a Latin-American pediatric population, dupilumab not only improves clinical outcomes but also enhances quality of life for both children and their caregivers.

Moreover, this study supports dupilumab as an effective therapeutic alternative in non-experimental or real-world settings, where factors such as treatment adherence and comorbidities may influence outcomes.

### Strengths and limitations

The present study, the first conducted in a Latin-American population, demonstrated that, in addition to achieving a clinically significant improvement—confirmed by a 0.5-point increase in ACQ-7-IA scores—in the group of patients treated with dupilumab, symptom control substantially enhanced patients’ quality of life and, consequently, the quality of life of their caregivers. This improvement in HRQoL, as measured by internationally validated instruments (PAQLQ(S)-IA and PACQLQ), represents an important contribution to the national literature supporting the current trend of using biological therapy in patients who have been refractory to conventional treatment.

## Conclusions

Treatment with dupilumab was associated with early and sustained improvements in symptomatic control of moderate-to-severe type 2 asthma in pediatric patients, as well as in the quality of life of both patients and their caregivers.

The benefits observed in this study, evident from the first weeks of treatment and maintained over 1 year, support the use of biological therapies as an effective alternative in cases inadequately controlled with conventional therapeutic management.

## Authors’ contributions

RMT and VGU conceived and designed the study. JPG, MJRS, THH, JZV, DMGG, and LAHZ contributed to patient recruitment, clinical data collection, and follow-up. CAGN participated in data interpretation and provided specialized input in pediatric pulmonology. VGU and RMT conducted the statistical analysis. VGU, RMT, and JPG drafted the manuscript. All authors critically revised the manuscript for important intellectual content, approved the final version to be published, and agree to be accountable for all aspects of the work.

## Ethics statement

Written informed consent was obtained from all participants or their legal guardians.

## Confirmation of originality

This manuscript is original, has not been published before, and is not under consideration for publication elsewhere.

## Disclosure of the use of generative AI and AI-assisted technologies

The authors used generative AI-assisted tools (ChatGPT, OpenAI) only for language editing. The authors reviewed and edited the content, and take full responsibility for the final manuscript.

## Funding source

This research did not receive any specific grant from funding agencies in the public, commercial, or not-for-profit sectors.

## Declaration of competing interest

Dr. Víctor Gonzalez-Uribe has received honoraria for lectures, consulting, and/or travel support from GSK, Sanofi, AstraZeneca, Faes Farma, ALK, and AbbVie. All other authors declare that they have no conflicts of interest related to this work.
